# Correlation Between Immune Cell Infiltration and PD-L1 Expression and Immune-Related lncRNA Determination in Triple-Negative Breast Cancer

**DOI:** 10.3389/fgene.2022.878658

**Published:** 2022-03-31

**Authors:** Wenlin Yang, Zhen Qiu, Junjun Zhang, Xiao Zhi, Lili Yang, Min Qiu, Lihua Zhao, Ting Wang

**Affiliations:** ^1^ Department of Pathology, Affiliated Hospital of Jining Medical University, Jining Medical University, Jining, China; ^2^ Department of Laboratory, Affiliated Hospital of Jining Medical University, Jining Medical University, Jining, China; ^3^ Department of Thyroid Surgery, Affiliated Hospital of Jining Medical University, Jining Medical University, Jining, China

**Keywords:** triple-negative breast cancer, immune cell infiltration, PD-L1, tumor mutational burden, immune-related lncRNAs

## Abstract

As a key element of the tumor microenvironment (TME), immune cell infiltration (ICI) is a frequently observed histologic finding in people with triple-negative breast cancer (TNBC), and it is linked to immunotherapy sensitivity. Nonetheless, the ICI in TNBC, to the best of our knowledge, has not been comprehensively characterized. In our current work, computational algorithms based on biological data from next-generation sequencing were employed to characterize ICI in a large cohort of TNBC patients. We defined various ICI patterns by unsupervised clustering and constructed the ICI scores using the principal component analysis (PCA). We observed patients with different clustering patterns had distinct ICI profiles and different signatures of differentially expressed genes. Patients with a high ICI score tended to have an increased PD-L1 expression and improved outcomes, and these patients were associated with decreased tumor mutational burden (TMB). Interestingly, it was showed that patients with high TMB exhibited an ameliorated overall survival (OS) than patients with low TMB. Furthermore, TMB scores only affected the prognosis of TNBC patients in the low-ICI score group but not in the high group. Finally, we identified a new immune-related lncRNA (irlncRNA) signature and established a risk model for the TNBC prognosis prediction. In addition, the high-risk group was related to poor prognosis, a high infiltration level of plasma B cells, monocytes, M2 macrophages, and neutrophils and a low PD-L1 expression. Therefore, the characterization and systematic evaluation of ICI patterns might potentially predict the prognosis and immunotherapy response in TNBC patients.

## Highlights

1) Identification of TNBC patients by utilizing the ICI scoring system to predict the prognosis and immunotherapeutic response.
2) The correlation of ICI scores with the PD-L1 expression and TMB score.
3) Construction of a novel 13-irlncRNA signature to evaluate the prognosis of TNBC patients.

## Introduction

As a type of aggressive cancer, triple-negative breast cancer (TNBC) includes the estrogen receptor (ER), progesterone receptor (PR), and human epidermal growth factor receptor-2 protein (HER-2). TNBC takes up roughly 10–20% of all breast cancers, and it is generally related to unfavorable prognosis compared with non-TNBC (Kumar and Aggarwal, 2016). Since TNBC responds to neither hormonal therapy nor medications targeting Her-2 protein, chemotherapy and immunotherapy play a pivotal role in the treatment of TNBC (Lyons, 2019). Immunotherapy empowers the host’s natural immune system to fight against tumor cells by activating various immune cells such as macrophages and CD8 T cells (Muenst et al., 2016). Studies have shown promising results that immunotherapy can improve the prognosis of TNBC. For instance, a current meta-analysis has noted that the blockade of PD1/PD-L1 can significantly improve the pathological complete response rates in TNBC patients, especially in patients at a high risk of relapse (Tarantino et al., 2021). Numerous results have demonstrated that higher infiltration levels of T lymphocytes are observed in TNBC, and the TNBC patients have the most promising outcomes in single-agent cancer immunotherapy compared with other molecular subtypes (Sugie, 2018). Nonetheless, not all TNBC patients would be universally appropriate for immunotherapy. Therefore, it is urgently vital to look for novel bio targets which can aid in the precise pretreatment selection of patients.

High-density inflammatory cell infiltration is not an uncommon histologic finding in TNBC, especially in high-grade TNBC. Advances in the field of immunotherapy have rejuvenated intense investigations on the so-called tumor microenvironment (TME), which predominately consists of transformed cells, including immune and stromal cells. Many studies concerning TME have elucidated that tumor-infiltrating lymphocytes (TILs) are intimately implicated in metastasis, recurrence, therapeutic response, and even patient survival of TNBC. For instance, Oshi and colleagues have found that inflammation is associated with improved prognosis in TNBC patients, while worse outcomes are observed in other types of breast cancer (Oshi et al., 2020). TNBC cells have also been observed to secrete interleukin (IL)-4, IL-10, and other factors (Laoui et al., 2011) to promote tumor-associated macrophage polarization toward M2, which is associated with fast tumor progression (Sousa et al., 2015). By comparison, increased TILs are linked with the increased disease-free survival (DFS) in TNBC (Tomioka et al., 2018), which may be related to escalated response to immunotherapy. However, given the intimate and complex interaction between tumor cells and various inflammatory cell infiltrates, it is insufficient to use single populations of inflammatory cells for predicting prognostication. Instead, characterization of the overall immune cell landscape would probably offer more valuable information. In addition, recent studies have focused on constructing the irlncRNA signature to predict the response of immunotherapy in many cancers, such as bladder cancer (Zhang L et al., 2020), hepatocellular carcinoma (Hong et al., 2020), clear cell renal cell carcinoma (Sun et al., 2020), and breast cancer (Shen et al., 2020). However, no studies have attempted to construct an irlncRNA risk model in TNBC patients.

In our current work, we analyzed the biological information obtained from next-generation sequencing to elucidate the gene expression profiles in TNBC and characterize the intra-tumoral landscape of the infiltrated immune cells. In addition, we demonstrated that TNBC could be categorized into two discrete subgroups with distinct outcomes based on the infiltration pattern of immune cells. We also established the immune cell infiltration (ICI) scoring system for predicting the prognosis and immunotherapeutic response. Finally, we identified a novel 13-irlncRNA signature and established a risk model to evaluate TNBC prognosis, as well as the correlation between clinicopathologic variables and the PD-L1 level or tumor mutational burden (TMB) score.

## Materials and Methods

### Discovery Cohort and Validation Cohort

The discovery cohort contained 435 TNBC samples from three available datasets (TCGA program and GSE33926 and GSE103091 datasets). The reads per kilobase of exon per million reads mapped (RPKM) data of 146 TNBC samples were acquired from TCGA (The Cancer Genome Atlas). The FPKM values were converted into TPMs to eliminate statistical biases inherent in the FPKM measure (Wagner et al., 2012). The microarray datasets (51 cases from GSE33926 and 238 cases from GSE103091) were from the GEO datasets (Gene Expression Omnibus). The corresponding array annotation files were adopted to annotate data, and genes represented by multiple probes were collapsed by averaging. The expressions of 13,723 genes were obtained after the three databases got merged and normalized. The “Combat” algorithm was applied to adjust the batch effect from multiple batches of microarray experiments (Johnson et al., 2007). Normalized expression matrices of the METABRIC program were obtained from cBioPortal for Cancer Genomics (http://www.cbioportal.org/). A total of 209 cases of basal-like breast cancer were set as the validation cohort.

### The Unsupervised Clustering of Tumor-Infiltrating Immune Cells

The CIBERSORT R package (Newman et al., 2015) was applied to evaluate the percentages of the 22 TILs by gene expression profiles, and 1,000 permutations were performed. As a newly developed algorithm, ESTIMATE (Yoshihara et al., 2013) was for inferring the fraction of immune and stromal cells, and their scores were determined to assess the infiltration levels of the cells in each sample. The ICI pattern of each TNBC sample was used to conduct hierarchical agglomerative clustering. The ConsensusClusterPlus R package was applied to conduct unsupervised clustering (Yu et al., 2012).

### Kaplan–Meier Plotter for Survival Analysis

OS (overall survival) analysis got conducted by the Kaplan–Meier plotter, and the log-rank test was for evaluating the differences between distinct subgroups. A *p*-value < 0.05 of the log-rank test was regarded as the statistical significance.

### Identification of DEGs Concerning the ICI Phenotype

The samples were categorized into the ICI clusters, and DEGs (differentially expressed genes) about ICI patterns were identified based on the optimal cutoff (FDR < 0.05 and |fold-change| > 1) through the limma R package.

### Functional Enrichment Analysis for DEGs

GO (gene ontology) analyses containing the BP (biological process), MF (molecular function), and CC (the cellular component), as well as KEGG (Kyoto Encyclopedia of Genes and Genomes) enrichment analyses, were performed using the R module profiler package to reveal the different biological pathways in gene signature groups A and B.

### Construction of High- and Low-ICI Score Groups

Each patient was categorized by unsupervised clustering based on the DEG values. ICI gene signature A was given to DEG values that were favorably related with the cluster signature, whereas signature B was given to DEG values that was negatively associated with the cluster signature. By using PCA (principal component analysis), the primary element became the signature score by the calculation formula as follows: ICI score (Zhang X et al., 2020) = *∑*PC1A- *∑*PC1B.

### GSEA in High- and Low-ICI Score Groups

GSEA was carried out through the GSEA software (Gene Set Enrichment Analysis, 4.0.1 version, http://software.broadinstitute.org/gsea/index.jsp). GSEA (Subramanian et al., 2005) examined a group of related genes that are highly up- or downregulated in a predetermined phenotype, allowing big gene sets to be broken down into smaller, more coherent sets, such as those that reflect a specific route.

### Construction of the irlncRNA Prognostic Model

To acquire irlncRNAs, 2,483 immune-related genes were obtained from the ImmPort database, and the correlation analysis was applied through the standards of correlation coefficients > 0.4 and *p* < 0.001. Subsequently, univariate and multivariate Cox regressions, as well as Lasso regression, were performed to establish a risk model. The AUC (area under the curve) values were computed, and the ROC (receiver operating characteristic) curve was plotted. The samples were categorized into high- and low-risk groups based on the best cutoff value. KMA was conducted to evaluate the OS between the two categories.

### Correlation of the Risk Score and ICI

Seven common methods, containing xCell, EPIC, quanTIseq, CIBERSORT-ABS, MCPcounter, TIMER, and CIBERSORT, were adopted to assess the ICI scores. The Wilcoxon signed-rank test and Spearman test were for the different analysis between distinct risk groups and immune infiltration statuses, and the correlation coefficients were calculated.

### Statistical Analyses

All data were processed by R software (version 4.0.3). The statistical analysis between the two categories was determined through the Wilcoxon rank-sum test, and the Kruskal–Wallis test was adopted for over two categories. The coefficient was calculated with Spearman correlation analysis. A Chi-square test was performed for the relationship between TMB and ICI scores. A *p* < 0.05 indicated the statistical significance.

## Results

### The Characterization of ICI in the Discovery and Validation Cohorts of TNBC

**FIGURE 1:**
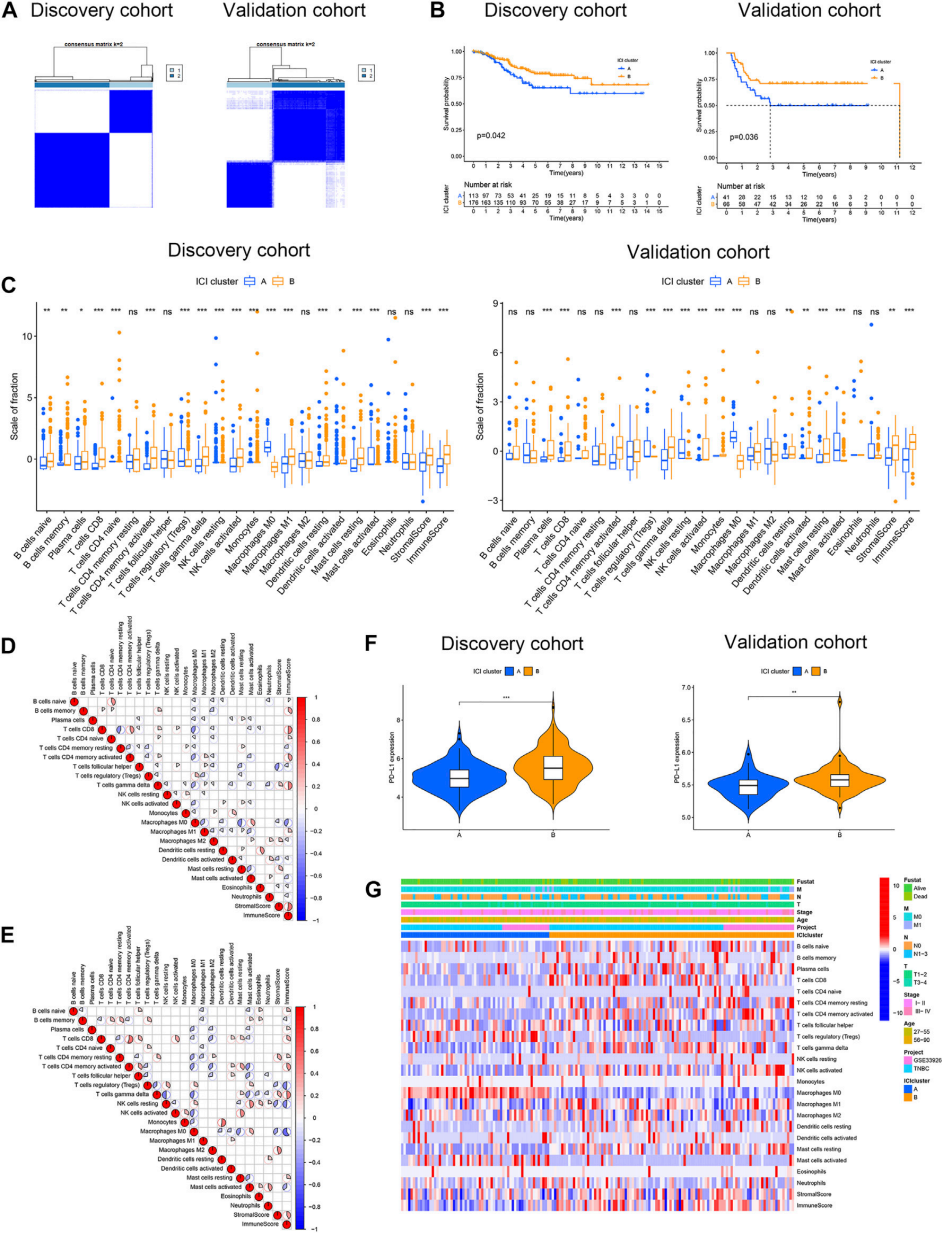
Characterization of ICI in two cohorts of TNBC. **(A)** Two independent ICI subtypes were determined through the ConsensusClusterPlus R package in the discovery and validation cohorts. **(B)** OS curves of two ICI subtypes in two cohorts. The log-rank test showed *p* = 0.042 and *p* = 0.036 in the discovery and validation cohorts. **(C)** Estimation of the fraction of 22 immune cells, as well as immune and stromal scores in ICI clusters A and B of two cohorts **(D,E)** Interaction of 22 immune cells, as well as immune and stromal scores, in the discovery cohort **(D)** and validation cohort **(E)**. **(F)** Different PD-L1 levels between ICI clusters A and B in two cohorts. **(G)** Unsupervised clustering of ICI with distinct clinical phenotypes in TCGA-TNBC and GSE33926 datasets. *p < 0.05; **p < 0.01; ***p < 0.001; and ****p < 0.0001.

CIBERSORT and ESTIMATE algorithms were combined to evaluate the infiltration level of immune and stromal cells in TNBC samples. In total, 435 cases were merged as the discovery cohort. A total of 209 cases of basal-like breast cancer from the METABRIC program were obtained as the validation cohort. The ConsensusClusterPlus R package was employed for unsupervised clustering to divide patients into different groups. Two distinct ICI subtypes were identified by unsupervised clustering (Figure 1A) with obviously different OS in both cohorts (log-rank test: p = 0.042, discovery cohort; p = 0.036, and validation cohort, Figure 1B) revealing that ICI cluster A had an improved prognosis compared with ICI cluster B. To further expound on the inherent divergences of the two subtypes, we compared 22 immune cell compositions, as well as immune and stromal scores. The infiltrations of only regulatory T cells (Tregs), resting NK cells, M0 macrophages, and activated mast cells were increased in ICI cluster A of the discovery and validation cohorts. The ICI cluster B was featured with high infiltration levels of the majority of rest immune cells, such as naive B cells, memory B cells, plasma, CD8 T cells, naive CD4 T cells, activated memory CD4 T cells, delta gamma T cells, and resting mast cells in the discovery cohort, and the immune and stromal scores were also greater. The result of the validation cohort conformed to the discovery cohort, except for naive B cells, memory B cells, naive CD4 T cells, and M1 macrophages (Figure 1C). Moreover, the correlation coefficient heatmap of the discovery cohort (Figure 1D) displayed a significant relation among immune cells, such as an obvious positive association between naive B cells and naive CD4 T cells, M0 macrophages and activated mast cells, and resting dendritic cells and eosinophils, and a marked negative correlation between CD8 T and resting memory CD4 T cells, and M0 macrophages and resting mast cells. Various immune cells, such as delta gamma T cells, were positively related to the Immune Score, while M0 macrophages were negatively correlated with the Immune Score, and helper follicular T cells were negatively correlated with the Stromal Score, which conformed to the results of the validation cohort (Figure 1E). Furthermore, the Wilcoxon rank-sum test was performed to analyze the PD-L1 expression in the two ICI subtypes, and the outcomes demonstrated the PD-L1 level was significantly greater in ICI cluster B compared with cluster A in both cohorts (Figure 1F). Figure 1G illustrates the unsupervised clustering of ICI with abundant clinical information in TCGA-TNBC and GSE33926 datasets.

### Determination of the Immune Gene Subtype in the Discovery Cohort of TNBC

**FIGURE 2:**
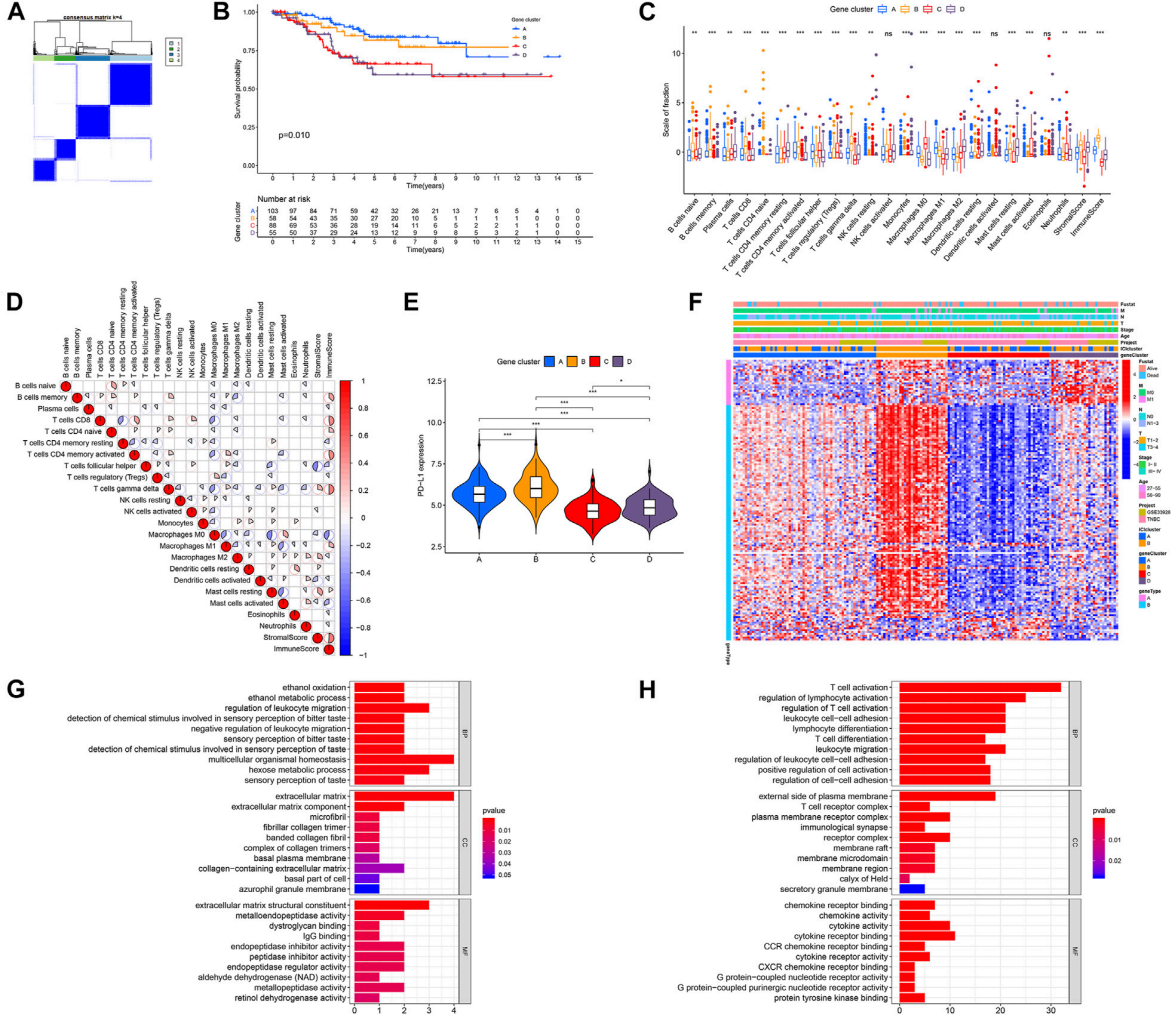
Identification of immune gene subtypes in the discovery cohort of TNBC. **(A)** Unsupervised clustering was performed, and the discovery cohort was divided into four gene clusters **(A–D)**. **(B)** KMA for four gene clusters, *p* = 0.010. **(C)** Fraction of 22 tumor-inﬁltrating immune cells, as well as immune and stromal scores, among four gene clusters. **(D)** Interaction of 22 immune cells, as well as immune and stromal scores, in the discovery cohort of TNBC. **(E)** PD-L1 expression levels in the four gene clusters. **(F)** Heatmap of DEGs in four gene clusters with distinct clinicopathological characteristics. **(G,H)** GO analysis of gene signatures A **(G)** and B **(H)**. *p < 0.05; **p < 0.01; and ***p < 0.001.

A total of 149 DEGs were obtained with the limma R package using the cutoff criteria of FDR <0.05 and |fold-change|> 1. Based on these DEGs, unsupervised clustering was performed, and the discovery cohort was divided into four genomic clusters (A, B, C, and D) (Figure 2A). Kaplan–Meier analysis (KMA) showed that clusters A and B were correlated with a significantly favorable prognosis, while clusters C and D were associated with a poor prognosis (Figure 2B). The difference in ICI was significant in the four-gene clusters, except for eosinophils, activated NK, and dendritic cells (Figure 2C). Gene clusters A and B were featured with a high Immune Score, while gene clusters C and D had the opposite results. The correlation coefficients between 22 immune cells and the Immune Score or Stromal Score were determined by correlation analysis (Figure 2D). We also found that the PD-L1 level was dramatically related to distinct gene clusters. Gene clusters A and B had a higher PD-L1 expression than gene clusters C and D (Figure 2E). Furthermore, we integrated the expressions of DEGs and clinical-pathological characters with gene clusters A–D. The heatmap is shown in Figure 2F. Next, we detached DEGs as gene signatures A and B that were positively or negatively associated with gene clusters, respectively. To remove the superfluous genes, the Boruta algorithm was performed, and 134 DEGs were selected to conduct further study. Finally, GO enrichment analysis showed different pathways between gene signatures A and B, indicating that distinct gene clusters were involved in different BPs. Figure 2G (gene signature A) and 2H (gene signature B) show the top 30 enriched pathways, revealing that gene signature A was enriched in multicellular organismal homeostasis, the extracellular matrix, and extracellular matrix structural constituent pathways, and gene signature B was related to the external side of the plasma membrane, T-cell activation, and cytokine receptor binding pathways.

### TNBC High- or Low-Score Groups According to ICI Scores

**FIGURE 3:**
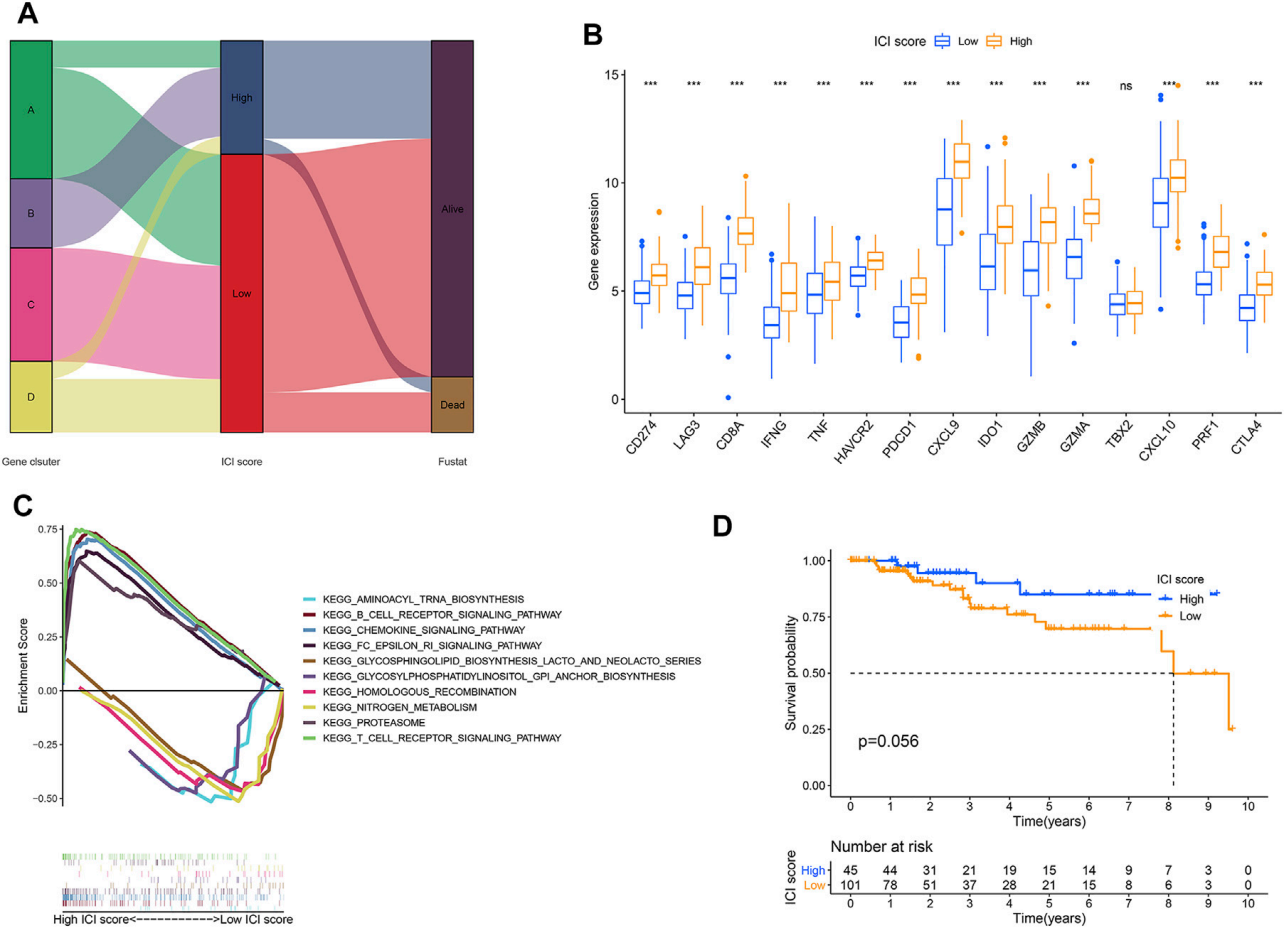
TNBC high- or low-score groups according to ICI scores. **(A)** Alluvial diagram of ICI gene cluster distribution in the four-gene groups with distinct ICI scores and survival states. **(B)** Expressions of immune checkpoint and immune activation relevant genes between two groups. **(C)** GSEA analysis in high- and low-ICI score groups. **(D)** KMA for two groups in the TCGA-TNBC cohort. *p* = 0.056.

According to gene signatures A and B, we used PCA to obtain ICI scores A and B of each TNBC patient. Figure 3A showed the allocation of patients in the four-gene clusters, as well as the ICI score and survival state. To compare immune conditions between high- and low-ICI score groups, we selected 15 immune-related genes, including CD274, IDO1, PDCD1, LAG3, HAVCR2, and CTLA4, which acted as immune checkpoint-related genes, and CD8A, IFNG, TNF, CXCL9, GZMA, GZMB, TBX2, CXCL10, and PRF1, which served as immune activation-related genes. We performed the Wilcoxon rank-sum test and found that all of these genes had higher expressions in the high-ICI score group than the low-ICI score group, except that TBX2 had no statistical significance (Figure 3B). Furthermore, we performed GSEA and found that different pathways were associated with the high- and low-ICI groups, respectively. Figure 3C showed the top five pathways with the most significant difference, indicating that a high ICI score was related to T-cell receptor, B-cell receptor, chemokine, fc_epsilon_ri signaling pathways, and proteasome, whereas the low-ICI score group was enriched with glycosylphosphatidylinositol gpl anchor biosynthesis, aminoacyl tRNA biosynthesis, nitrogen metabolism, homologous recombination, and glycosphingolipid biosynthesis lacto and neolacto series pathways. To compare the OS of the two groups, we performed the KMA and log-rank test. Although no obvious difference was detected (p = 0.056), the TNBC patients of the TCGA cohort had a better prognosis in the high-ICI score group (Figure 3D).

### A Combination of ICI and TMB Scores Can Be Used to Predict the Prognosis in TNBC

**FIGURE 4:**
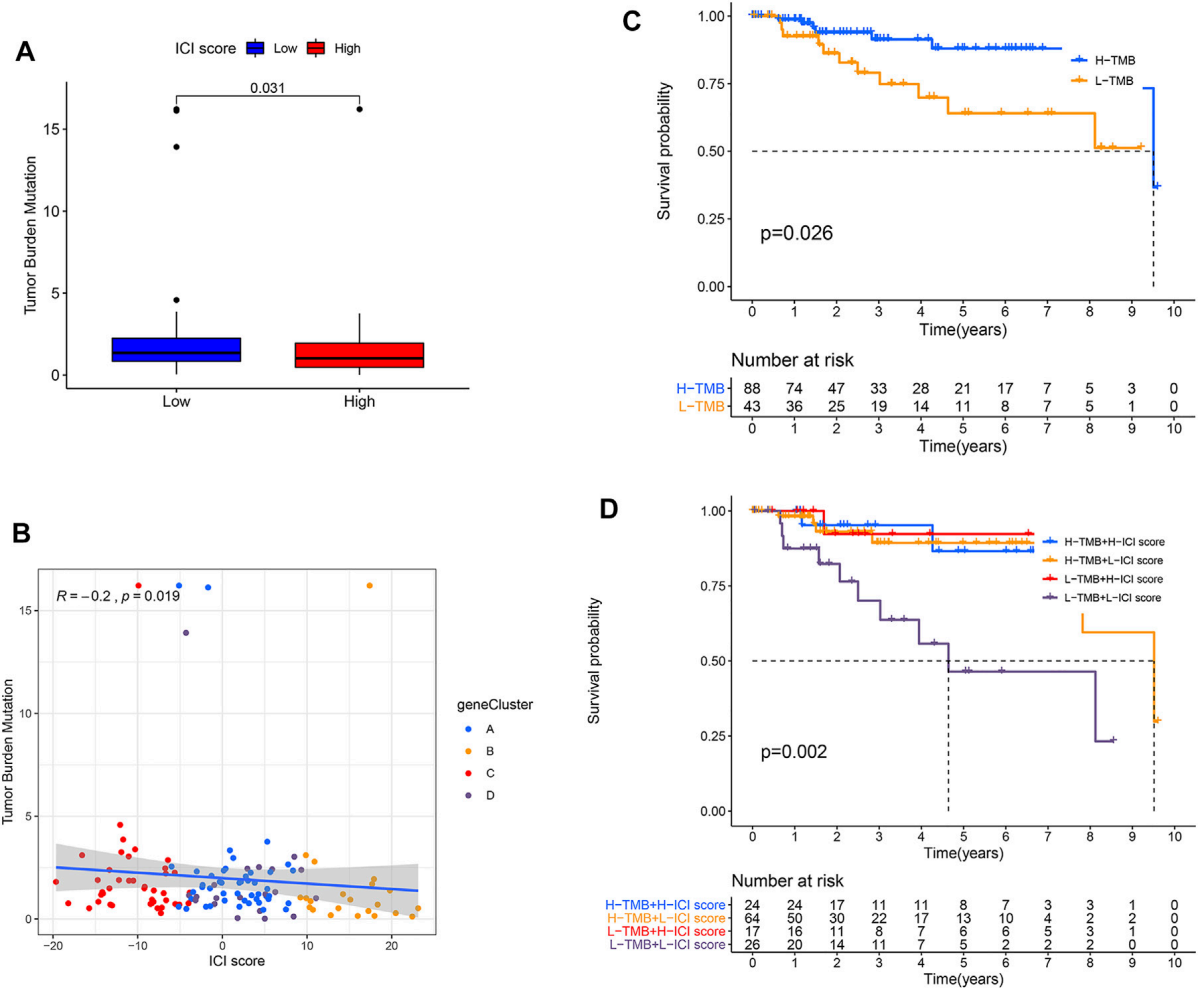
Combination of ICI and TMB scores to predict prognosis in TNBC. **(A)** Compared TMB level between two ICI score subgroups. *p* = 0.031. **(B)** Correlation between ICI and TMB scores in four gene clusters. Spearman test, correlation coefficient (R) =−0.2, *p* = 0.019. **(C)** OS curves for high- and low-TMB groups of the TCGA-TNBC cohort. *p* = 0.026. **(D)** KMA for patients in the TCGA-TNBC cohort was stratiﬁed by both TMB and ICI scores. *p* = 0.002.

Increasing evidence has shown that TMB has an important effect on the immunotherapeutic response of cancer patients (Rizvi et al., 2015). To reveal the inherent correlation between TMB and ICI score groups, we assessed the TMB levels of TNBC patients in the TCGA cohort. Our data revealed that patients had a dramatically lower level of TMB in the high-ICI score group (*p* = 0.031, Figure 4A). We further ensured TMB and ICI scores had an obvious negative relation (Figure 4B). Interestingly, the patients with high TMB had a better OS than individuals with low TMB (Figure 4C). Considering the prognosis value of ICI and TMB scores, we further assessed the antagonistic impacts of these scores on the prognosis stratification of TNBC patients. It showed the low-TMB + low-ICI score group had the worst prognosis, while the high-TMB + high-ICI score group and low-TMB + high-ICI score group had a favorable prognosis. We also observed no remarkable difference existed between high-TMB and low-TMB patients in the high-ICI score group, whereas the high-TMB group displayed a remarkably favorable prognosis compared with the low-TMB group in the low-ICI score group (Figure 4D). All the findings demonstrated the combination of ICI and TMB scores might better predict the immunotherapeutic response and outcomes in TNBC patients.

### Construction of the irlncRNA Prognostic Model

**FIGURE 5:**
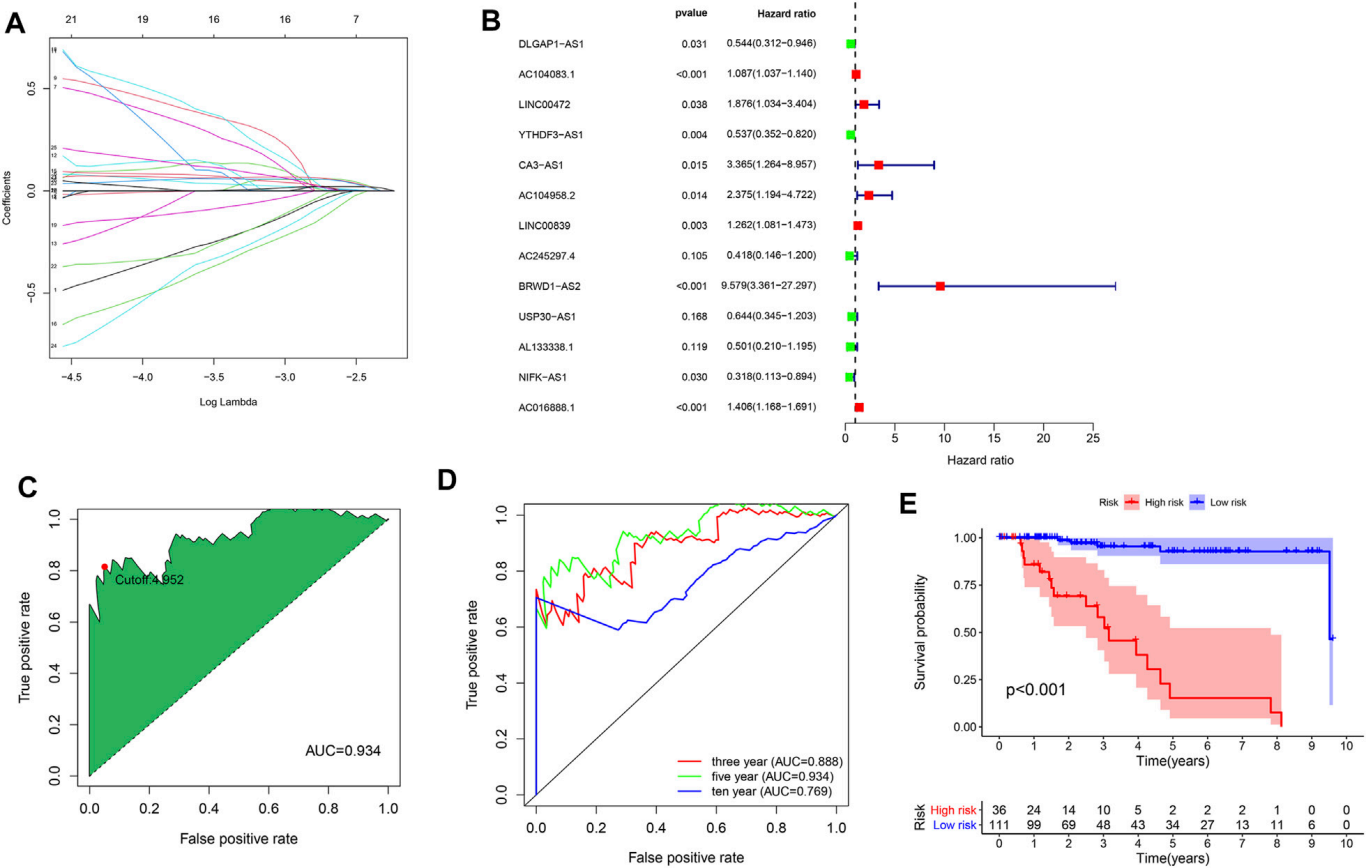
Construction of the irlncRNA prognostic model. **(A)** Lasso regression analysis. **(B)** Forest map of identified irlncRNAs by Cox proportional hazard regression. **(C)** ROC curve was plotted with the AUC value and optimal cutoff value. **(D)** The 3-, 5- and 10-year ROC curves were mapped with AUC values. **(E)** KMA between the high- and low-risk groups, *p* < 0.001.

To establish an irlncRNA risk model, we obtained 2,483 immune-related genes from the ImmPort database. By performing the correlation analysis, we identified 1,370 irlncRNAs. Integrated with survival information, univariate Cox regression analysis was adopted to obtain survival-related irlncRNAs. Lasso analysis (Figure 5A) and multivariate Cox regression analysis (Figure 5B) were further applied to establish a prognosis signature. The expressions of 13 irlncRNAs were for calculating the risk score by the following formula: risk score = Exp (DLGAP-AS1) × (-0.61) + Exp (AC104083.1) × (0.08) + Exp (LINC00472) × (0.63) + Exp (YTHDF3-AS1) × (−0.62) + Exp (CA3-AS1) × (1.21) + Exp (AC104958.2) × (0.86) + Exp (LINC00839) × (0.23) + Exp (AC245297.4) × (−0.87) + Exp (BRWD1-AS2) × (2.26) + Exp (USP30-AS1) × (−0.44) + Exp (AL133338.1) × (−0.69) + Exp (NIFK-AS1) × (−1.15) + Exp (AC016888.1) × (0.34). Next, we plotted a 5-year ROC curve and calculated the AUC, and the maximum inﬂection point was accepted as the cutoff point by the AIC values (Figure 5C). The 3, 5, and 10-year ROC curves were also mapped with high AUC values over 0.75 (Figure 5D). KMA suggested a significantly better prognosis in the low-risk group than the high-risk group (Figure 5E, log-rank test, *p* < 0.001).

### Correlation of the Risk Score With Clinicopathologic Variables, Immune Cells, PD-L1 Expression, and TMB Score

**FIGURE 6:**
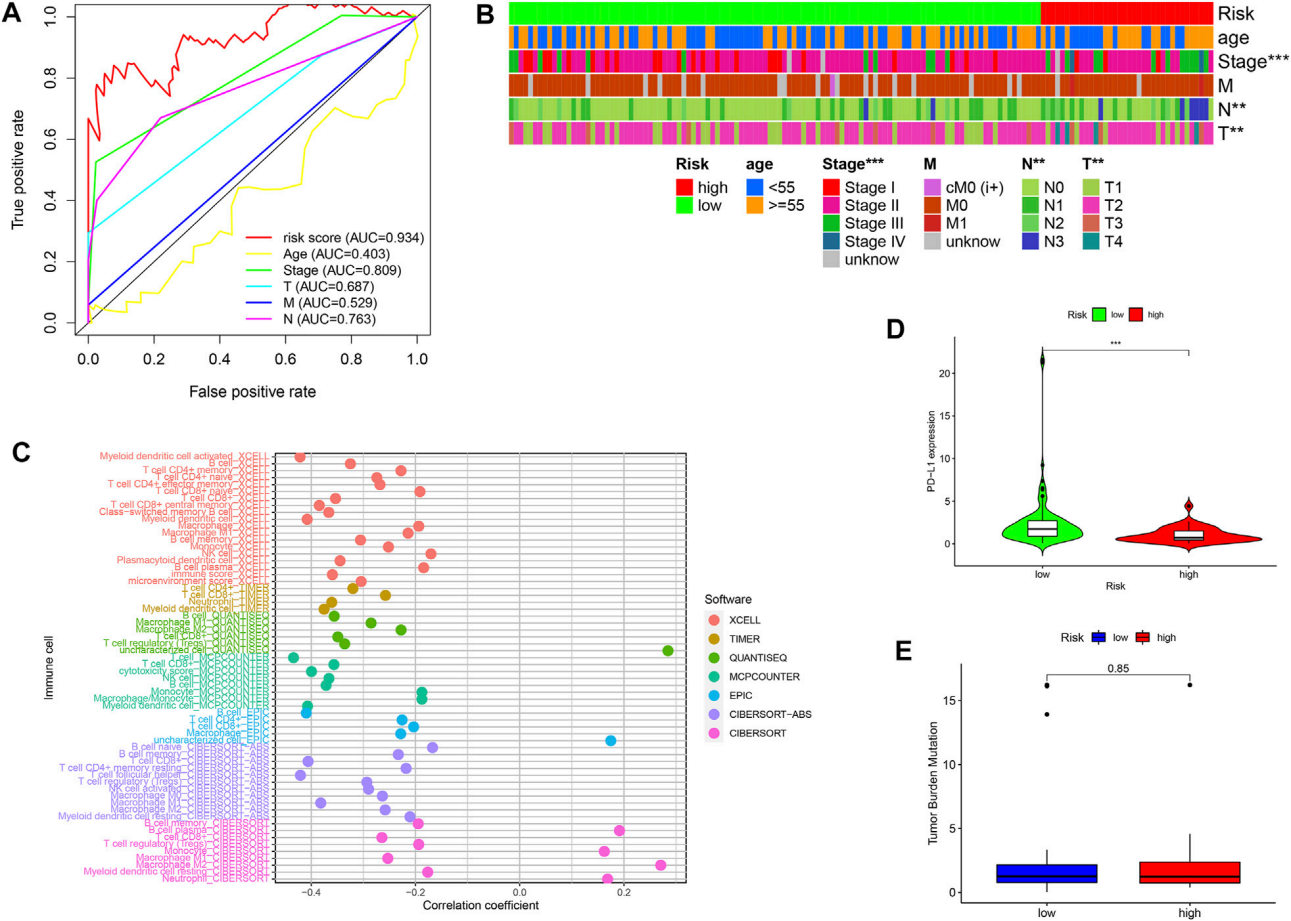
Correlation of the risk score with clinicopathological variables, immune cells, PD-L1 expression, and TMB scores **(A,B)** A comparison of 5-year ROC curves **(A)** and correlation analysis **(B)** between risk scores and traditional clinicopathologic variables. **(C)** Spearman analysis between risk scores and ICI with seven common methods. **(D)** Different PD-L1 expression levels in the high-risk and low-risk groups. **(E)** Compared TMB level in the high-risk and low-risk groups. *p < 0.05; **p < 0.01; and ***p < 0.001.

Furthermore, we compared the AUC values between the risk score and traditional clinicopathologic variables, including clinical, T, M, N, stages, and age. Figure 6A showed that the risk score with the highest AUC value was compared with other variables. Moreover, the chi-square tests revealed the risk score was associated with clinical, N and T stages (Figure 6B). To study the mechanism between irlncRNA risk scores and ICI, the Spearman test was conducted, revealing that high-risk score was negatively related to most immune cells, except for plasma B, monocytes, M2 macrophages, and neutrophil cells (Figure 6C). In addition, the PD-L1 expression was lower in the high-risk group than that of the low-risk group (Figure 6D). However, there was no statistical difference in the TMB score between the two groups (Figure 6E). These findings indicated the irlncRNA risk score was related to the PD-L1 level but not with the TMB score.

## Discussion

In our current work, we successfully constructed the ICI scoring system and demonstrated that it could be used as a robust biomarker for predicting the immunotherapeutic response in a cohort consisting of 644 TNBC patients. Although immunotherapy is highly effective in suppressing tumor growth and improving patient life quality, it is limited by the high cost. This conundrum is further aggravated by the fact that only a minority of patients favorably receive immunotherapy. As a result, it is vital to accurately identify people who might benefit from immunotherapy.

To devise novel immune-modulatory strategies to deal with TNBC, we need to better understand the immune characteristics and profiles of TNBC. Herein, we categorized TNBC from a meta-cohort including 435 samples into ICI clusters A and B. The ICI cluster B, characterized by more infiltrations of CD4 T, CD8 T, B, activated NK cells, M1 macrophages, and high expression of immune checkpoint molecule PD-L1 showed improved OS, which was consistent with the previous studies (Wu et al., 2019; Tokumaru et al., 2020; O'Melia et al., 2021). Intense ICI has been reported to be present in as high as 48% of TNBC cases, especially in TNBC with the basal-like subtype (Harano et al., 2018). TNBC patients with high levels of immune infiltration, which are measured by immune signatures, show an improved OS (Iwamoto et al., 2011). In addition, for TNBC patients undergoing neoadjuvant chemotherapy, intense ICI is associated with a higher pathological complete response and a better outcome. A neoadjuvant GeparSixto trial has shown that a subset of TNBC patients with strong immunologic signals can hopefully benefit from the immunotherapy strategy (Denkert et al., 2015). Researchers have reported that PD-L1 is expressed in immune cells of 40–65% TNBC tissues (Beckers et al., 2016), and PD-L1 ( + ) tumors have a greater CD8 ( + ) T-cell infiltration compared with PD-L1 (−) tumors (Mittendorf et al., 2014). However, some PD-L1 (−) patients still obtain a clinical response with immune checkpoint inhibitors (Ribas and Hu-Lieskovan, 2016). Furthermore, only an objective response rate of 18.5% has been reported in TNBC patients in a recent phase Ib clinical trial of PD-L1 immune checkpoint inhibition (Nanda et al., 2016). Therefore, simple ICI alone is insufficient for the precise prediction of immunotherapeutic response.

Therefore, we hypothesized that a combination of data from the ICI model and immune-related signature would offer more information concerning individualized immunotherapy. We observed that ICI gene clusters A and B were linked with a favorable prognosis and a dramatically greater immune score than clusters C and D. In addition, there were increased infiltrations of B cells, CD8 T cells, activated memory CD4 T cells, and M1 macrophages cells in ICI gene clusters A and B. Therefore, patients categorized into clusters A or B might benefit from immunotherapy. In contrast, clusters C and D with higher infiltrations of plasma, resting memory CD4 T cells, M0 cells, and M2 macrophages, exhibited an immune-cold phenotype. Plasma cell-predominant breast cancer has been reported as an independently predicted value for worse OS and DFS (Wei et al., 2016). In contrast, research has shown that aforementioned median densities of CD38^+^ plasma cells are associated with a better DFS but not OS (Yeong et al., 2018). Presently, we revealed that TNBC patients with high infiltrations of plasma cells had a poor outcome. M0 and M2 macrophages are strongly associated with a poor outcome, contributing to cell migration in breast cancer (Ali et al., 2016; Tu et al., 2021). Furthermore, we divided DEGs into ICI gene signatures A and B to obtain tumor subtype-speciﬁc biomarkers, which have been well studied to enhance the outcome prediction (Callari et al., 2016). Compatible with previous studies, ICI gene signature A was linked to multicellular organismal homeostasis and extracellular matrix pathways, which have been noted to portend improved survival for patients with breast cancer (Roy and Walsh, 2014).

Through GSEA, we revealed that genes involved in the T- and B-cell receptor, proteasome, and chemokine signaling pathways were related to the high-ICI score group. However, glycosylphosphatidylinositol gpl anchor biosynthesis, aminoacyl tRNA biosynthesis, nitrogen metabolism, and homologous recombination pathways were connected with the low-ICI score group, which have been rarely reported in cancer. The KMA showed the high-ICI score group exhibited a better prognosis compared with the low-ICI score group, although there was no statistical significance (p = 0.056). Furthermore, increasing evidence demonstrates TMB is related to immunotherapeutic response in breast cancer (Thomas et al., 2018) and other types of cancer (Zhang et al., 2019; Lv et al., 2020). For instance, Romualdo et al. have shown that a high TMB has a relation with a longer OS in metastatic TNBC patients treated with anti-PD-1/L1 therapies (Barroso-Sousa et al., 2020). In line with this result, our study indicated TNBC patients with a higher TMB exhibited a better OS. We also noted that TMB was remarkably increased in patients with low ICI scores, indicating a significant negative relation between TMB and ICI scores. The combined use of TMB and ICI scores suggested that patients with a low-TMB + low-ICI score had the worst prognosis. We also found that TMB scores had no prognostic value in the high-ICI score group, whereas the high-TMB + low-ICI score group had a favorable prognosis compared with the low-TMB + low-ICI score group. This finding suggested that the combination of ICI and TMB scores could improve the prediction of outcomes to immunotherapy.

Furthermore, we identified a novel 13-irlncRNA signature to design a risk model to assess TNBC prognosis. Among these irlncRNAs, LINC00472 acts as a tumor suppressor and predictive marker in breast cancer (Shen et al., 2015a). The high expression of LINC00472 has been correlated with ER-positive, low-grade breast cancer, and favorable molecular subtypes (Shen et al., 2015b). The cell experiment has confirmed that LINC00472 suppresses the phosphorylation of NF-kB through binding to IKKβ in breast cancer (Wang et al., 2019). The nuclear lncRNA Linc00839 is upregulated in chemoresistant breast cancer cells, and its overexpression enhances Myc and activates the PI3K/AKT signaling pathway, thus facilitating proliferation, invasion, and migration, as well as leading to a poor prognosis in breast cancer (Chen et al., 2020). By calculating the AUC values, we verified the risk model could better forecast the 3-, 5-, and 10-year survival rate than the traditional clinicopathologic characteristics. Moreover, a high-risk score marked with poor prognosis and low PD-L1 had a positive relation with infiltrations of plasma B cells, monocytes, M2 macrophages, and neutrophils, which was consistent with the ICI score.

## Conclusion

Collectively, we comprehensively evaluated the ICI landscape of TNBC with biological information obtained from next-generation sequencing with computational algorithms. Characterization of the ICI landscape served to elucidate the complex and dynamic anti-/pro-tumor immune response regulation in TNBC. Moreover, the ICI patterns were negatively correlated with TMB in TNBC. Therefore, the characterization and systematic evaluation of the ICI patterns in combination with TMB scores in TNBC might potentially serve to identify candidate patients for optimal individualized immunotherapy. The combination of ICI and TMB scores might function as a potentially effective biomarker for immunotherapeutic response prediction in TNBC patients. Furthermore, a novel 13-irlncRNA signature was determined and applied to conduct a risk model to accurately predict TNBC prognosis.

## Data Availability

The original contributions presented in the study are included in the article/Supplementary Material, further inquiries can be directed to the corresponding authors.

## Abbreviations

AIC, Akaike information criterion; AUC, area under the curve; BP, biological processes; CC, cellular components; DFS, disease-free survival; ER, estrogen receptor; ESTIMATE, Estimation of STromal and immune cells in MAlignant Tumor tissues using Expression data; GO, Gene Ontology; GSEA, gene set enrichment analysis; HER-2, human epidermal growth factor receptor-2 protein; ICI, immune cell infiltration; IL, interleukin; irlncRNA, immune-related lncRNA; KEGG, Kyoto Encyclopedia of Genes and Genomes; KMA, Kaplan–Meier analysis; MF, molecular functions; OS, overall survival; PCA, principal component analysis; PR, progesterone receptor; ROC, receiver operating characteristic; TILs, tumor-infiltrating lymphocytes; TME, tumor microenvironment; TMB, tumor mutational burden; TNBC, triple-negative breast cancer; TPMs, transcripts per kilobase million.
